# Erythropoietin – a potential tool in the treatment of depressive disorders?

**DOI:** 10.1192/j.eurpsy.2022.1440

**Published:** 2022-09-01

**Authors:** G. Simões, S. Jesus, R. Silva

**Affiliations:** Baixo Vouga Hospital Centre - EPE, Psychiatry And Mental Health Department, Aveiro, Portugal

**Keywords:** Depression, depressive disorders, Erythropoietin, Pharmacology

## Abstract

**Introduction:**

Depression is one of the leading causes of psychiatric disability across the globe because of its high prevalence and chronic, treatment resistant and recurrent nature. Erythropoietin (EPO), well known for its effects on blood cells, has also a key role in neuroprotection and cognitive function.

**Objectives:**

The authors aim to explore the potential of EPO to treat depressive disorders (DD) and related cognitive dysfunction.

**Methods:**

A literature research was conducted on PubMed starting from the MeSH terms: “Erythropoietin” and “Depressive Disorders”. The results selected for our analysis corresponded to investigations using EPO based on an adult population with DD.

**Results:**

The research provided 14 results, of which 9 met the defined criteria. Different types of studies with variable samples were considered, including randomized clinical trials (RCTs) and a systematic review. Overall, despite records of reduction in depression symptomatology and increased quality of life, evidence does not demonstrate statistically significant reductions in depression severity through the use of EPO in the treatment of DD. However, several RCTs examined its effect on cognitive performance, founding effective improvements in memory, verbal recall and recognition. The underlying potential mechanisms and the current limitations in the use of EPO, and of the available studies are analysed and discussed.

**Conclusions:**

Although EPO does not appear to be effective treating depression, it may play a role in improvement of deficits in memory and executive function. Larger RCTs evaluating its potential use are needed, in order to move towards better clinical practice, quality of life and functional reintegration of these patients.

**Disclosure:**

No significant relationships.

